# The Potential of Iodine and Iron Double-Fortified Salt Compared with Iron-Fortified Staple Foods to Increase Population Iron Status

**DOI:** 10.1093/jn/nxaa204

**Published:** 2021-02-15

**Authors:** Richard F Hurrell

**Affiliations:** Institute of Food, Nutrition and Health, ETH Zurich, Zurich, Switzerland

**Keywords:** iron fortification efficacy, salt, cereals, sensory changes, consumption patterns, industry landscape

## Abstract

The potential of double-fortified salt (DFS) to improve population iron status is compared with the potential of iron-fortified wheat flour, maize flour, rice grains, and milk products. The potential for a positive impact on iron status is based on reported efficacy studies, consumption patterns, the extent of industrialization, and whether there are remaining technical issues with the fortification technologies. Efficacy studies with DFS, and with iron-fortified wheat flour, maize flour, and rice, have all reported good potential to improve population iron status. Iron-fortified milk powder has shown good impact in young children. When these foods are industrially fortified in modern, automated facilities, with high-level quality control and assurance practices, high-quality raw materials, and a wide population coverage, all vehicles have good potential to improve iron status. Relative to other fortification vehicles, fortification practices with wheat flour are the most advanced and iron-fortified wheat flour has the highest potential for impact in the short- to medium-term in countries where wheat flour is consumed as a staple. Liquid milk has the least potential, mainly because an acceptable iron fortification technology has not yet been developed. Maize is still predominantly milled in small-scale local mills and, although the extruded rice premix technology holds great promise, it is still under development. Salt has a proven record as an excellent vehicle for iodine fortification and has demonstrated good potential for iron fortification. However, technical issues remain with DFS and further studies are needed to better understand and avoid color formation and iron-catalyzed iodine losses in both high- and low-quality salts under different storage conditions. There is currently a risk that the introduction of DFS may jeopardize the success of existing salt iodization programs because the addition of iron may increase iodine losses and cause unacceptable color formation.

## Introduction

Iron fortification of foods is a key strategy to address iron deficiency (ID) and iron deficiency anemia (IDA). The most common iron fortification vehicles used in national programs are wheat and maize flours, rice grains, and milk products and, to this small number, can now be added double-fortified salt (DFS). This article reviews the potential of DFS, as compared with iron-fortified wheat, maize, rice, or milk products, to improve population iron status. The estimated potential is based on the reported efficacy of the respective fortification technologies in long-term feeding studies in women and children, and on the national consumption patterns and industrial landscapes of the different food vehicles. Remaining technical issues are also considered including sensory changes that could decrease consumption, and the possibility that adding iron to iodized salt may jeopardize the success of current salt iodization programs. The relative costs of fortification, the ability of the consumer to afford and accept the price increase of the fortified food, the consumer's access to the fortified foods, and the need for multimicronutrient fortification of a single vehicle have not been considered in this review.

Efficacy studies were identified from past published systematic reviews of randomized controlled iron fortification trials (1) and DFS efficacy studies (2) and a search of PubMed to end of November 2018 for randomized, controlled, iron fortification efficacy studies of wheat flour, maize flour, milk, and DFS. The selected efficacy studies had monitored iron status using hemoglobin (Hb) and a range of specific iron status biomarkers. The difficulties in monitoring iron status, particularly in low- and-middle-income countries (LMICs) with widespread infections, are discussed in the introductory article of this supplement (3).

Most studies in the current review judged that the efficacy of an iron fortification intervention was established when improved iron status was demonstrated by ≥1 of the following parameters: increased serum ferritin (SF) [or plasma ferritin (PF)]; increased body iron stores (BIS); decreased transferrin receptor (TfR) and decreased zinc protoporphyrin; together with a decrease of ID and IDA. Some studies had monitored iron status with Hb alone. This is not recommended because, although Hb is decreased with ID, it can also be decreased by infections, inflammation, and hemoglobinopathies (3), common in many LMICs. Inflammation can also influence iron status biomarkers (3) and most efficacy studies, considered in the current review, that were made in populations with a high prevalence of inflammation either excluded subjects with inflammation from the SF analyses or adjusted SF values based on inflammation biomarkers (4).

The selected efficacy studies were further evaluated in relation to whether the increased daily iron intake during the study period would be expected to meet the estimated daily iron needs of the subjects. In order to ensure efficacious iron fortification, the increased daily iron intake needed by women and children in LMICs, consuming a 10% iron bioavailability diet, is estimated to be 7 mg Fe/d as ferrous sulfate, adjusted according to the relative bioavailability of the iron fortification compound used (5, 6).

It should be noted that efficacy studies are well-planned, randomized feeding studies in which defined numbers of subjects at risk of ID (young women or children) consume iron-fortified test meals or non-iron-fortified control meals. The feeding is supervised, the additional iron intake calculated, and, at the end of the study, the iron status in the test group is compared with that in the control group. This is of course an ideal situation, but not the real-life situation which can be monitored by an effectiveness study. Such studies have been used to monitor national programs where the iron status of subsections of the population (young women and children) is measured at baseline, when the fortification program is introduced, and again after several years as part of the national diet (7). Such effectiveness studies are not controlled. They are influenced by the good manufacture, quality control, distribution, coverage, and price of the fortified food, consumer acceptance of any sensory changes, as well as the efficacy of the fortification technology, consumption patterns, and the industrial landscape.

## Efficacy

### Wheat and maize flour

**Table 1** shows efficacy studies in women and children who consumed iron-fortified wheat or maize flours fortified with different iron compounds. These studies indicate that national programs of wheat flour fortification that follow WHO (6) recommendations and fortify with ferrous sulfate or sodium iron ethylenediaminetetraacetic acid (NaFeEDTA) should, in the absence of widespread infections and inflammation, improve iron status at the population level. Efficacy studies with electrolytic iron have given variable results but indicate that programs should be effective provided that widespread infections and inflammation are absent. Fortification with ferrous fumarate would be expected to be as effective as ferrous sulfate, although this has not been confirmed in efficacy studies with cereal staples. Similarly, iron fortification of maize flours should be as efficacious as wheat flours, but again there are few efficacy studies to confirm this.

#### Ferrous sulfate

There are 4 efficacy studies where ferrous sulfate was added to wheat flour to provide from 7.1 to 10.3 mg fortification iron per day to young children, adolescents, or women of child-bearing age in Thailand, China, Kuwait, and Morocco. All studies resulted in a significant improvement in iron status (all *P* < 0.05) (Table 1). In the Kuwait study, ferrous sulfate encapsulated with partially hydrogenated soy bean oil significantly improved iron status based on increased SF and increased BIS (both *P *< 0.001) (8).

#### Ferrous fumarate

Neither of the efficacy studies with ferrous fumarate (Table 1) improved iron status (*P* > 0.05). The WHO recommendations (6) for ferrous fumarate are the same as for ferrous sulfate (7 mg Fe/d), and the low fortification amount (2.5 mg Fe/d) of the bread South African schoolchildren consumed over 8 mo probably explains their unchanged iron status at the end of the trial (13).

The Hansen et al. (9) study in young Swedish women, however, provided 8.6 mg Fe/d as ferrous fumarate and would have been expected to improve iron status. Because there was a significant decrease in SF in the control group but not the test group, it could be argued that fortification iron had a positive effect on iron status; however, this is far from impressive. Although whole-grain rye bread was used to provide the fortification iron, the authors report a low phytic acid concentration in the bread due to prolonged fermentation. The most likely explanation for this disappointing result is that there were few study subjects and the study duration was only 5 mo, instead of the recommended 6–9 mo (5).

#### NaFeEDTA

Table 1 also shows the results from 4 efficacy studies with wheat flour, and 1 efficacy study with whole-maize porridge fortified with NaFeEDTA. The efficacy studies with white wheat flour [in Morocco (10) and China (11)] and with whole-grain atta flour in India (12) provided 4.1–6.7 mg Fe/d and were highly efficacious. The study in South Africa, providing only 1.3 mg Fe/d to children in brown bread, as would be expected (6), had no effect on iron status (13).

**TABLE 1:** Efficacy studies with wheat-, maize-, or rye-based foods fortified with different iron compounds and monitored with a combination of iron status biomarkers^1^

Reference, country	Subjects^2^	Duration	Fortified food	Additional iron^3^	Inflammation^4^	Impact^5^
Ferrous sulfate/ferrous fumarate^6^						
Zimmermann et al. (15), Thailand	330 women aged 18–50 y, 3 groups	35 wk	Low extraction wheat snacks	10.3 mg/d	Not measured	SF and BIS increased, TfR decreased, ID decreased (34%–7%)
Sun et al. (11), Northern China	400 children aged 11–18 y, with IDA, 4 groups	6 mo	70% extraction wheat flour	8.8 mg/d	Not measured	SF increased, TfR decreased, IDA decreased (100%–40%)
Biebinger et al. (8), Kuwait	279 women aged 18–35 y, 3 groups	5.5 mo	Low extraction wheat biscuits	7.1 mg/d	Not measured	SF and BIS increased, TfR decreased
Bouhouch et al. (10), Morocco	457 children aged 4–10 y, 4 groups	28 wk	Low extraction wheat biscuits	6.7 mg/d	>40%	SF^7^ and BIS increased, TfR decreased, ID (38%–5%) and IDA (14%–3%) decreased
Hansen et al. (9), Sweden	46 women aged 20–30 y, 2 groups	5 mo	Whole-rye bread	8.6 mg/d	Not measured	No change in SF (or Hb) with added iron; but decrease in SF in controls
van Stuijvenberg et al. (13), South Africa	361 children aged 6–11 y, 4 groups	34 wk	80% extraction wheat bread	2.5 mg/d	3%–5%	No change in SF (or Hb)
NaFeEDTA						
Bouhouch et al. (10), Morocco	457 children aged 4–10 y, 4 groups	28 wk	Wheat flour biscuits	6.7 mg/d	>40%	SF and BIS increased, TfR decreased, ID (42%–6%) and IDA (18%–2%) decreased
Muthayya et al. (12), India	401 children aged 6–15 y, 4 groups	7 mo	Whole-grain wheat chapatti	4.1 mg/d	Inflammation excluded	SF and BIS increased, TfR and ZPP decreased, ID (63%–20%) and IDA (19%–10%) decreased
Sun et al. (11), Northern China	400 children aged 11–18 y, with IDA, 4 groups	6 mo	70% extraction wheat flour	5.9 mg/d	Not measured	SF increased, TfR decreased, IDA decreased (100%–1%)
Andang'o et al. (14), Kenya	516 children aged 3–8 y, 4 groups	5 mo	Whole-maize porridge	4.6 mg/d	5%–9% at baseline, negligible at end^8^	SF increased, ID (18%–3%) and IDA (12%–2%) decreased)
van Stuijvenberg et al. (13), South Africa	361 children aged 6–11 y, 4 groups	34 wk	80% extraction wheat bread	1.3 mg/d	3%–5%	No impact on SF, ID, or IDA
Electrolytic iron						
Zimmermann et al. (15), Thailand	330 women aged 18–50 y, 3 groups	35 wk	Low extraction wheat snacks	10.3 mg/d	Not measured	SF and BIS increased, TfR decreased, ID decreased (28%–11%)
van Stuijvenberg et al. (16), South Africa	160 children aged 6–11 y, 3 groups	7 mo	80% extraction wheat bread	2.3 mg/d	Not measured	No change in SF
Sun et al. (11), Northern China	400 children aged 11–18 y, with IDA, 4 groups	6 mo	70% extraction wheat flour	20 mg/d	Not measured	No change in SF, IDA decreased (100%–60%)
Andang'o et al. (14), Kenya	516 children aged 3–8 y, 4 groups	5 mo	Whole-maize porridge	4.5 mg/d	5%–9% at baseline, negligible at end^8^	No change in SF, ID, or IDA
van Stuijvenberg et al. (13), South Africa	361 children aged 6–11 y, 4 groups	34 wk	80% extraction wheat bread	4.5 mg/d	3%–5%	No change in SF, ID, or IDA
Rohner et al. (17), Côte d'Ivoire	591 children aged 6–14 y, 8 groups	6 mo	Low extraction wheat biscuits	9 mg/d	13%–26%	No change in Hb, SF,^9^ TfR, ZPP, or ID

1 BIS, body iron stores; CRP, C-reactive protein; Hb, hemoglobin; ID, iron deficiency; IDA, iron deficiency anemia; NaFeEDTA, sodium iron ethylenediaminetetraacetic acid; SF, serum ferritin; TfR, transferrin receptor; ZPP, zinc protoporphyrin.

2 All studies were randomized and placebo controlled with approximately the same number of subjects in each group. Subjects aged 3–18 y were of both sexes.

3 The additional iron consumed per day over the study duration was calculated by dividing the combined amount of iron consumed on test days (excluding weekend days and school holidays if the test meal was not consumed) by the total number of days in the study duration.

4 Inflammation based on CRP, or on CRP plus α-1-acid-glycoprotein [Bouhouch et al. (10) and Rohner et al. (17)].

5 Reported increases or decreases of iron status parameters relative to control were statistically significant (at least *P *< 0.05), except for Andang'o et al. (14) where statistical differences were not reported. More precision on the statistical significance is given in the text. No changes in iron status parameters are reported when differences relative to control were *P *> 0.05.

6 Zimmermann et al. (15), Sun et al. (11), and Bouhouch et al. (10) fortified with ferrous sulfate, Biebinger et al. (8) fortified with encapsulated ferrous sulfate, and Hansen et al. (9) and van Stuijvenberg et al. (13) with ferrous fumarate.

7 SF corrected (4).

8 Subjects treated for malaria before end-point measurements.

9 Cutoff for ID taken as SF >30 μg/L instead of >12 μg/L.

The study with maize flour was in Kenya with 4- to 8-y-old children who consumed maize porridge (14). Because the study was conducted in a malaria-endemic area, the children were treated for malaria infection before blood draws so as to avoid the influence of inflammation on iron biomarkers. ID and IDA (based on Hb, SF, and TfR) were reported to decrease with an additional 4.6 mg Fe/d. Although this study is more difficult to interpret, owing to its relatively short length, and presumably high inflammation during the feeding period, it would seem to support the 2009 WHO (6) recommendation of 4.6 mg Fe/d as NaFeEDTA.

#### Electrolytic iron

In 2009, the WHO (6) estimated that 14 mg Fe/d as electrolytic iron powder would be needed to achieve efficacy. This fortification amount is more or less supported by the 5 efficacy studies with wheat flour and the single study with maize flour (Table 1). Providing 10 and 20 mg Fe/d as electrolytic iron to women and adolescents in Thailand (15) and Northern China (11) improved iron status, whereas providing 2.3–4.5 mg Fe/d to children in South Africa (16) and Kenya (14) had no effect on iron status.

The study of Rohner et al. (17) in Côte d'Ivoire is a cause of some concern because it provided 9 mg Fe/d to 6- to 14-y-old children for 6 mo and found no benefit to iron status based on SF, TfR, and Hb. SF was not adjusted, despite widespread infection, but ID was defined as SF <30 μg/L instead of the usual cutoff of <12 μg/L used in the absence of inflammation. The Estimated Average Requirement (EAR) for 6- to 13-y-old children consuming a 10% iron bioavailability diet is 10.6 mg Fe/d (3), and 50% EAR for 6- to 13-y-old children would be 5.3 mg Fe/d. Assuming electrolytic iron is half as well absorbed as ferrous sulfate, the recommended iron intake from electrolytic iron for the 6- to 14-y age group would be a little over 10 mg Fe/d, not so far removed from the 9 mg Fe/d provided in the study.

The Rohner et al. (17) study was in a malaria-endemic area where inflammation was high, and where iron absorption would be expected to be decreased. However, this is not the only explanation for no impact, because in a recent study in a similar malaria-endemic area in Côte d'Ivoire, 1- to 3-y-old children who consumed a maize/soy complementary food fortified with 5.8 mg Fe/d as NaFeEDTA and ferrous fumarate for 9 mo increased their SF concentrations considerably (18). A poorer solubility of electrolytic iron during digestion in malnourished Ivorian children could be an explanation.

The Andang'o study in Kenya (14) was also in a malaria-endemic area and 1 group of 3- to 8-y-old children in this study were provided with electrolytic iron at 4.5 mg Fe/d for 5 mo. This amount of iron is well below 50% of the EAR for electrolytic iron for this age group, which would be 7.4 mg/d for a 10% iron bioavailability diet (3) and would explain why there was no impact on iron status.

### Rice

In total, 8 efficacy studies have been published with iron-fortified rice (**Table 2**). In all studies, the extruded rice premix technology (3, 19) was used to manufacture the iron-fortified rice. In all studies, ferric pyrophosphate (FPP) was the iron fortification compound, either as micronized ground ferric pyrophosphate (MGFP) or as micronized dispersible ferric pyrophosphate (MDFP). Some studies manufactured the premix rice grains from fortified rice flour using the hot extrusion technology (70–110°C) and others used the cold extrusion technology (30–40°C) (19). Recent iron absorption studies (20) indicate that the lower iron bioavailability from hot extruded premix rice than from cold extruded premix rice is compensated for by decreased iron losses when the premix rice is cooked in excess water. It would appear that hot extruded premix rice is becoming the technology of choice.

#### MGFP

There are 6 efficacy studies with MGFP (Table 2). Three studies, which provided an additional iron intake greater than the recommended 14 mg/d for FPP (5), all showed good improvements in iron status with increases in SF, and decreases in ID from 80% to 25% (*P *< 0.01) in 6- to 13-y-old Indian schoolchildren (hot extrusion technology) (21), from 33% to 14% (*P *< 0.05) in 5- to 11-y-old Indian schoolchildren (cold extrusion technology) (22), and from 65% to 25% (*P *> 0.01) in 6- to 24-mo-old Brazilian infants (cold extrusion technology) (23).

Studies providing <14 mg Fe/d have reported an inconsistent influence on iron status, however. Thankachan et al. (24) (hot extrusion technology) provided 12.5 mg Fe/d in the test meals but, because these meals were not consumed on Sundays or during the extended school holidays, the mean amount consumed by the 6- to 12-y-old Indian children over the 6-mo study was only 8 mg/d, and this amount had no influence on status. Although Hussain et al. (25) reported an increase in SF (*P *< 0.05) and a useful decrease in ID by providing 7 mg Fe/d to 4- to 8-y-old Indian children, this can be explained by the much lower iron requirement of this age group (3).

The most recent study in Cambodia (26), with 6- to 16-y-old children, compared 2 types of hot extrusion with cold extrusion, and provided from 7.4 to 10.5 mg Fe/d. The children's ferritin concentrations were highly elevated owing to inflammation and changes in adjusted SF during the feeding were inconsistent, with increases in SF in the 2 hot extrusion groups (*P *< 0.001) but no change in SF in the cold extrusion group. The high inflammation could explain the unchanged Hb and BIS, the unexpected increase in TfR (*P *< 0.001) after 6 mo in 1 group who consumed the hot extruded rice, and the inconsistent SF. Other studies do not indicate that cold extruded iron-fortified rice is less efficacious than hot extruded and, on the contrary, Hackl et al. (20) recently reported a 60% higher iron absorption in young women from cold extruded rice than from hot extruded rice fortified with regular (nonmicronized) FPP.

There have been 2 further studies with MGFP and cold extrusion; however, these studies used Hb alone as the iron status biomarker and the results should be treated with caution. Parker et al. (27) provided 13 mg Fe/d for 7 mo to 7- to 11-y-old schoolchildren in Burundi and reported no change in Hb (*P *> 0.05), presumably owing to the high inflammation. Nogueira Arcanjo et al. (28) provided 56 mg Fe on 1 single day per week to 10- to 23-mo-old Brazilian infants (8 mg Fe/d for 18 wk) and decreased anemia prevalence from 28% to 11% (*P *< 0.05).

#### MDFP

There have been 2 efficacy studies with MDFP (Table 2). Both studies used cold extrusion and both reported reasonably good improvements in iron status. Angeles-Agdeppa et al. (29) in the Philippines provided 9 mg Fe/d and reported a significant increase in PF (*P *< 0.05) and Hb (*P *< 0.05) in anemic schoolchildren, with anemia prevalence falling from 100% to 33%. Ferrous sulfate at the same fortification amount gave similar results but resulted in colored rice grains. Hotz et al. (30) provided 13 mg Fe/d and reported an increase in SF (*P *< 0.05) in young Mexican women after 6 mo feeding iron-fortified rice and a decrease in ID from 33% to 23% (*P *< 0.05).

**TABLE 2:** Efficacy studies with rice fortified with MGFP or MDFP (SunActive iron) and monitored with a combination of iron status parameters^1^

Reference, country	Subjects^2^	Duration	Fortified food^3^	Additional iron^4^	Inflammation^5^	Impact^6^
MGFP						
Moretti et al. (21), India	184 children aged 6–13 y, 2 groups	7 mo	Hot extruded premix rice	17 mg/d	Inflammation excluded	SF and BIS increased, TfR decreased, ID (80%–25%) and IDA (30%–15%) decreased
Beinner et al. (23), Brazil^7^	175 infants aged 6–24 mo, 2 groups	5 mo	Cold extruded premix rice	23 mg/d	Inflammation excluded	SF increased, ID decreased (65%–25%)
Radhika et al. (22), India	140 children aged 5–11 y, 2 groups	8 mo	Cold extruded premix rice	16 mg/d	Inflammation excluded	SF increased, ID decreased (33%–14%)
Thankachan et al. (24), India	258 children aged 6–12 y, 3 groups	6 mo	Hot extruded premix rice^8^	8 mg/d	Inflammation excluded	No change in SF, TfR, ZPP, or Hb
Hussain et al. (25), India	222 children aged 5–8 y, 6 groups	6 mo	Hot extruded premix rice	7 mg/d	Not measured	SF increased, ID decreased (82%-26%)
Perignon et al. (26), Cambodia^8^	2440 children aged 6–16 y, 5 groups	6 mo	Hot and cold extruded premix rice	7.4–10.5 mg/d	>40%	No change in Hb and BIS, SF and TfR increased
MDFP						
Hotz et al. (30), Mexico	201 women aged 18–49 y, 2 groups	6 mo	Cold extruded premix rice	13 mg/d	Not measured	PF and BIS increased, TfR decreased, ID (33%–23%) and anemia (21%–4%) decreased
Angeles-Agdeppa et al. (29), Philippines	180 children aged 6–9 y, 3 groups	6 mo	Cold extruded premix rice	9 mg/d	Not measured	PF increased, anemia decreased (100%–33%)

1 BIS, body iron stores; CRP, C-reactive protein; Hb, hemoglobin; ID, iron deficiency; IDA, iron deficiency anemia; MDFP, micronized dispersible ferric pyrophosphate; MGFP, micronized ground ferric pyrophosphate; PF, plasma ferritin; SF, serum ferritin; TfR, transferrin receptor; ZPP, zinc protoporphyrin.

2 All studies were randomized and placebo controlled with approximately the same number of subjects in each group. Subjects aged 6 mo–16 y were of both sexes.

3 Iron-fortified rice is produced by mixing regular nonfortified rice grains with iron-fortified premix rice grains manufactured from fortified rice flour by hot (70–110°C) or cold (30–40°C) extrusion (19).

4 The additional iron consumed per day over the study duration was calculated by dividing the combined amount of iron consumed on test days (excluding weekend days and school holidays if the test meal was not consumed) by the total number of days in the study duration.

5 Inflammation based on CRP, or on CRP plus α-1-acid-glycoprotein [Perignon et al. (26)].

6 Reported increases or decreases of iron status parameters relative to control were statistically significant (at least *P *< 0.05). More precision on the statistical significance is given in the text. No change in iron status parameters is reported when differences relative to control were *P *> 0.05.

7 Not placebo controlled; iron-fortified rice was compared with ferrous sulfate drops (10 mg Fe) with similar impact.

8 Premix rice with multimicronutrients.

MGFP and MDFP have thus both shown useful efficacy but, although MDFP may have a higher fractional absorption than MGFP in some food vehicles (and may allow lower fortification amounts), MGFP is preferred owing to its much lower cost. In addition, studies indicate that iron absorption from regular FFP can approach that of ferrous sulfate on the addition of trisodium citrate and citric acid to hot extruded rice (31). This is an important new development and appears to be due to the formation of more soluble iron citrate complexes during the processing and cooking. It would appear that nonmicronized FPP could replace MGFP for premix rice fortification.

### Milk

There is ample evidence that commercial powdered infant formula based on cow milk fortified with ferrous sulfate and ascorbic acid is highly efficacious in improving the iron status of infants from 6 to 18 mo (32–34). Likewise, there is good evidence that public health interventions providing young children with iron-fortified spray-dried cow milk (with ascorbic acid) improve iron status. Villalpando et al. (35) provided 5.8 mg Fe/d as ferrous gluconate in reconstituted whole cow milk to 10- to 30-mo-old Mexican children for 6 mo, and reported substantial decreases in ID (68%–28%, *P *< 0.001) and anemia (41%–12%, *P *< 0.001) as compared with the control children. The beneficial effects on iron status were subsequently confirmed in a much larger effectiveness study (36). Likewise, Sazawal et al. (37) provided 1- to 4-y-old Indian children with 9.6 mg Fe/d as ferrous sulfate in 3 servings of reconstituted, multimicronutrient-fortified milk powder per day for 1 y. There were significant increases in Hb (*P *< 0.001) and SF (*P *< 0.001) in the test group receiving the fortification iron compared with the control group, and the children receiving iron-fortified milk powder had an 88% lower risk of IDA. Powdered milk has not been foreseen as an iron fortification vehicle for all age groups but specifically to target ID in young children. Several Latin American countries have iron-fortified powdered milk programs targeted at young children.

Liquid milk has proven more difficult to fortify with iron (3) and only 1 efficacy study has been reported (38). In this study, 6- to 14-y-old Saudi children consumed 1 L of flavored liquid milk per day (as 5 × 200-mL packages) providing 6 mg Fe/d as ferrous bisglycinate (FBG) for 3 mo. Hb values increased significantly (*P *< 0.0001) and the prevalence of anemia fell from 24% to 8%. However, SF values increased in the girls only (*P *< 0.01). The study was weakened by having no control group.

### DFS

Larson et al. (39) conducted a systematic review and meta-analysis of 20 published DFS efficacy studies and reported an overall positive effect of DFS consumption on iron status, with increased Hb and SF concentrations and decreased IDA. Instead of a meta-analysis approach, the following section focuses in detail on each study's DFS formulation, the iron compound that was used, and any potential factors that may have affected the impact of DFS on anemia or iron status indicators. Together with the approach of Larson et al. (39), this evaluation provides a broader picture of the iron compounds used in DFS development.

DFS has been developed mainly in India over the last 40 y (3). During that time, a range of iron compounds and fortification technologies have been evaluated so as to overcome color changes and prevent increased iodine losses that can occur upon the addition of iron to salt. Studies are presented in relation to the biomarkers that were used to monitor impact (**Table 3**): either using Hb alone (mostly the early studies) or using a range of iron status biomarkers (more recent studies).

In general, the mostly early studies using Hb alone as the iron status biomarker showed no improvement or only modest improvements in iron status. This includes the efficacy studies where the iron complexing agent sodium hexametaphosphate (SHMP) was added to ferrous sulfate to prevent color changes and iodine losses (DFS Type 2) (40), and studies with nonencapsulated ferrous fumarate (DFS Type 1a) (41). In contrast, the more recent efficacy studies that monitored impact using a range of specific iron status biomarkers showed good improvements in iron status for encapsulated ferrous fumarate (EFF) (DFS Type 1b), ferrous sulfate encapsulated with partially hydrogenated soy bean oil (DFS Type 4), and MGFP (DFS Type 5).

#### DFS Type 1a, nonencapsulated ferrous fumarate

Asibey-Berko et al. (41) in Ghana measured the efficacy of nonencapsulated ferrous fumarate (1 mg Fe/g salt) in DFS containing encapsulated iodine (Table 3). The salt provided 15- to 45-y-old women with ∼10 mg Fe/d for 8 mo so would be expected to be efficacious; however, there was no change in anemia prevalence in the women presumably owing to the presence of inflammation.

#### DFS Type 1b, EFF

Andersson et al. (42) used DFS Type 1b with 2 mg Fe/g and provided 12 mg Fe/d to 5- to 15-y-old Indian children for 10 mo. There were increases in SF, Hb, and BIS (all *P *< 0.001), the ID prevalence decreased from 52% to 35% (*P *< 0.001), and IDA prevalence decreased from 12% to 4% (*P *< 0.001) (Table 3). In a second study by Haas et al. (43), female Indian tea pickers aged 18–55 y consumed Type 1b DFS (1.1 mg Fe/g). The DFS provided 10–13 mg Fe/d and, although there was no change in anemia prevalence, SF increased (*P *< 0.05) and ID decreased from 26% to 9% (*P *< 0.05) (Table 3). Inflammation, and/or deficiencies in vitamin B-12 and folate, could have been important causes of the anemia in this setting because α-1-acid-glycoprotein was elevated in >20% of the women and another 20% also presented with macrocytic anemia. As evidence of the improved iron status, Wenger et al. (44) reported improvements in the perceptual, attentional, and mnemonic performance of the female tea pickers consuming the DFS.

The efficacy studies with DFS Type 1b (Table 3) provided 12 mg Fe/d (42, 43). This is higher than the recommended amount for nonencapsulated ferrous fumarate which is 7 mg Fe/d (5). Whereas DFS has often been fortified to provide 1 mg Fe/g salt (2), the efficacy studies with DFS Type 1b provided 1–2 mg Fe/g salt. Clearly it would be an advantage to decrease the fortification amount in relation to lower cost and less potential for sensory changes and iodine losses; however, this also depends on salt consumption.

#### DFS Type 1c, EFF

This is the current version of EFF that is used in program settings. As yet, there are no efficacy studies with this new form of EFF which was developed in order to decrease color changes in the stored salt. It has a similar combination of coatings to DFS Type 1b but is manufactured by hot extrusion technology instead of fluidized bed granulation (3). Other differences from DFS Type 1b are that it has semolina flour added before extrusion and does not contain SHMP (51).

**TABLE 3:** Iron efficacy studies with DFS monitored using Hb alone or a combination of iron status biomarkers^1^

Reference, country	Subjects^2^	Duration	DFS	Additional iron^3^	Inflammation^4^	Impact^5^
Monitored using Hb alone						
Nadiger et al. (45), India	546 children aged 5–15 y, 2 boys and 2 girls groups^6^	12 mo	FePO4 plus Na H sulfate	15 mg/d	Not measured	Anemia decreased in boys (54%–19%) and girls (16%–3%)
Working Group Report (46), India	*c*.12,000 subjects of all ages, groups^7^	12 mo	FePO4 plus Na H sulfate	15 mg/d	Not measured	Anemia decreased in all test groups
Asibey-Berko et al. (41), Ghana	182 women aged 15–45 y, 3 groups	8 mo	DFS 1a	10 mg/d	Not measured	No change in anemia
Reddy and Nair (47), India	947 children aged 6–15 y, 4 groups	9 mo	DFS 2	Salt (1 mg Fe/g) provided	Not measured	No change in anemia
Monitored using a combination of iron status biomarkers						
Zimmermann et al. (48), Morocco	377 children aged 6–15 y, 2 groups	9 mo	DFS 4	10 mg/d	Not measured	SF increased, TfR, ZPP, and IDA (35%–8%) decreased
Zimmermann et al. (49), Morocco	158 children aged 6–15 y, 2 groups	10 mo	DFS 5	18 mg/d	Not measured	SF and BIS increased, TfR, ZPP, and IDA (30%–5%) decreased
Wegmüller et al. (50), Côte d'Ivoire	123 children aged 5–15 y, 2 groups^8^	6 mo	DFS 5	10 mg/d	Inflammation excluded	No change in Hb; SF, TfR, and BIS increased
Andersson et al. (42), India	458 children aged 5–15 y, 3 groups	10 mo	DFS 5	12 mg/d	Not measured	SF and BIS increased, ZPP, TfR, ID (56%–34%), and IDA (15%–7%) decreased
Andersson et al. (42), India	458 children aged 5–15 y, 3 groups	10 mo	DFS 1b	12 mg/d	Not measured	SF and BIS increased, ZPP, TfR, ID (52%–35%), and IDA (12%–4%) decreased
Haas et al. (43), India	212 women aged 18–55 y, 2 groups	8 mo	DFS 1b	12 mg/d	>22%	No change in Hb; SF and BIS increased, TfR and ID (26%–9%) decreased

1 BIS, body iron stores; CRP, C-reactive protein; DFS, double-fortified salt; DFS 1a, double-fortified salt with nonencapsulated ferrous fumarate; DFS 1b, double-fortified salt with Nutrition International encapsulated ferrous fumarate; DFS 2, double-fortified salt with ferrous sulfate plus sodium hexametaphosphate; DFS 4, double-fortified salt with ferrous sulfate encapsulated with partially hydrogenated soybean oil; DFS 5, double-fortified salt with micronized ground ferric pyrophosphate; Hb, hemoglobin; ID, iron deficiency; IDA, iron deficiency anemia; SF, serum ferritin; TfR, transferrin receptor; ZPP, zinc protoporphyrin.

2 Unless otherwise stated, all studies were randomized and placebo controlled with approximately the same number of subjects in each group. Subjects aged 5–15 y were of both sexes.

3 The additional iron consumed per day over the study duration was calculated by dividing the combined amount of iron consumed on test days (excluding weekend days and school holidays if the test meal was not consumed) by the total number of days in the study duration.

4 Inflammation based on CRP (50), or on CRP plus α-1-acid-glycoprotein (43).

5 Reported increases or decreases of iron status parameters relative to control were statistically significant (at least *P *< 0.05). More precision on the statistical significance is given in the text. No change in iron status parameters is reported when differences relative to control were *P *> 0.05.

6 Study not randomized but made in 4 residential schools, with control schools having fewer than half the subjects that were in the test schools.

7 Study not randomized but made in 4 control and 4 test villages in different regions of India.

8 Study made in a malaria-endemic area.

Type 1c DFS uses a sophisticated encapsulation process to prevent color formation and iodine losses, and this encapsulation process may decrease iron absorption. Whereas the encapsulation of ferrous fumarate and ferrous sulfate with a single coating of hydrogenated oils is not thought to decrease their relative bioavailability (52), the Type 1c DFS formulation has multiple coatings with soy stearine, titanium dioxide, and hydroxy propyl methyl cellulose over a core of ferrous fumarate extruded with semolina flour. Hot extrusion itself has recently been shown to decrease iron absorption (20). As a first step, a stable isotope iron bioavailability study in human subjects consuming DFS Type 1c DFS would indicate if the capsule decreases iron absorption from ferrous fumarate. A more urgent need, however, is to demonstrate improved iron status in feeding studies with women or children consuming DFS Type 1c.

#### DFS Type 2, ferrous sulfate plus SHMP

Different ferrous sulfate formulations have been tested for efficacy and effectiveness (40, 53). Ferrous sulfate plus SHMP was suggested for fortification of the more highly refined salts and was tested (1 mg Fe/g salt) in a 2-y-long feeding study in a collection of Indian villages and in 4 residential schools (53). Daily salt intake in India was estimated to be 12–20 g/d with a mean of 15 g/d (54). The results from both studies were disappointing with no consistent increases in Hb in the test groups, and both increases and decreases in the Hb concentrations in the control groups. Malaria was endemic in some areas where the studies took place. More recent studies with ferrous sulfate and SHMP (Table 3), again using only Hb as the iron status biomarker, have also reported no impact on anemia prevalence in schoolchildren (47).

These efficacy studies with ferrous sulfate and SHMP provided >7 mg Fe/d and would be expected to improve iron status, yet, presumably because Hb alone was used as the iron status biomarker, evidence of efficacy was not demonstrated. In order to confirm the efficacy of DFS Type 2, the efficacy studies must be repeated with biomarkers, such as SF, that are specific to iron status.

#### DFS Type 3, ferrous sulfate plus various chelating agents

In Vinodkumar and Rajagopalan (55), residential schoolchildren in India consumed a multimicronutrient-fortified salt with iron added as ferrous sulfate. The ferrous sulfate was stabilized with malic acid and SHMP and sodium dihydrogen phosphate was added as an absorption enhancer. Iron status was monitored by Hb alone. Hb concentrations increased significantly (*P *< 0.05) by 0.6 g/dL after 1 y of feeding in the test group, with a small decrease (*P *< 0.05) in Hb in the control group. Salt intake was estimated at 10 g/d and the children consumed in the school ∼20 d/mo, indicating that a mean of ∼7 mg Fe/d was provided as ferrous sulfate over the 12-mo feeding period. Riboflavin and vitamins A and B-12 were in the multimicronutrient mixture added to the salt and could also have improved Hb.

#### DFS Type 4, ferrous sulfate encapsulated with partially hydrogenated soy bean oil

Zimmermann et al. (48) fortified DFS with ferrous sulfate encapsulated with partially hydrogenated soy bean oil (1 mg Fe/g salt). DFS was provided to families at the village level in Morocco and after 9 mo the iron status of 6- to 15-y-old children was greatly improved (Table 3). The children consumed 7–12 mg additional iron per day via the salt and after 9 mo IDA had decreased from 35% to 8% (*P *< 0.001). However, the DFS turned the salt a slight yellow color in the rainy season as the moisture content of the salt increased. This could perhaps have been prevented with the addition of SHMP or other iron-chelating agents.

#### DFS Type 5, MGFP

There are 3 DFS studies with MGFP (Table 3), all with 5- to 15-y-old children. The Zimmermann et al. (49) MGFP study in Morocco, as with their earlier study with DFS Type 4, reported an impressive improvement in iron status of the children after 10 mo feeding. The salt was fortified with 2 mg Fe/g and provided ∼18 mg additional iron/d. Providing this salt to Moroccan households improved all iron status biomarkers (*P *< 0.001) in children after 10 mo and decreased IDA in Moroccan schoolchildren from 30% at baseline to 5% after 10 mo (*P *< 0.001). The DFS was also an effective vehicle for iodine fortification and the improvement in iodine status reported compared favorably with that of iodine-fortified salt without added iron. Median urinary iodine concentration increased in both groups from a deficient level at baseline to a sufficient level at 10 mo. Thyroid volume and goiter prevalence also decreased significantly in both groups and, consistent with previous reports (56), the addition of iron to salt improved iodine efficacy. This is because iron is needed for the thyroperoxidase enzyme that incorporates absorbed iodine into thyroglobulin in the thyroid. Thyroid size and goiter prevalence were thus decreased to a greater extent in the DFS group than in the group receiving iodized salt without iron.

The Wegmüller et al. (50) study in Côte d'Ivoire was in a malaria-endemic area with high inflammation. The salt was fortified with 3 mg Fe/g and provided an additional 10 mg Fe/d. Considering the high inflammation present, it was not surprising that anemia prevalence was unchanged; however, despite the relatively low additional iron intake (10 mg instead of the recommended 14 mg Fe/d) and the shorter study duration, SF increased significantly (*P *< 0.05) and ID decreased from 100% to 52% (*P *< 0.01). In the most recent study, Andersson et al. (42) provided 12 mg Fe/d as MGFP in DFS containing 2 mg Fe/g in the breakfast and dinner meals of 5- to 15-y-old village children near Bangalore, India for 10 mo. Infections were reported to be low but inflammation was not reported. Iron status was significantly improved as demonstrated by increases (*P *< 0.01) in SF and BIS, and by decreases in ID prevalence from 56% to 35% (*P *< 0.001) and IDA prevalence from 15% to 7% (*P *< 0.05). However, iodine losses were high in the salt containing a high moisture content (1.8%).

#### Ferric orthophosphate plus sodium hydrogen sulfate

This formulation, although widely included in the early Indian studies, is not in current use and was not included in the different types of DFS described by Baxter and Zlotkin (57). In Nadiger et al. (45), Indian children in residential homes consumed salt fortified with 1 mg Fe/g as ferric orthophosphate (FOP) plus sodium hydrogen sulfate for 1 y (Table 3). Estimated iron intake from the salt (15 mg Fe/d) for 1 y increased Hb significantly in both boys and girls and decreased anemia prevalence from 53.7% to 19.4% (*P *< 0.001) in boys and from 15.5% to 3% (*P *< 0.001) in girls, with little change in the control groups. When the same formulation was consumed in large-scale feeding trials in different areas of India, with *c*.2000 subjects in both control and test groups, Hb concentrations in all test populations increased and there were significant reductions in the prevalence of anemia in men, women, and children in all areas (46).

**FIGURE 1:**
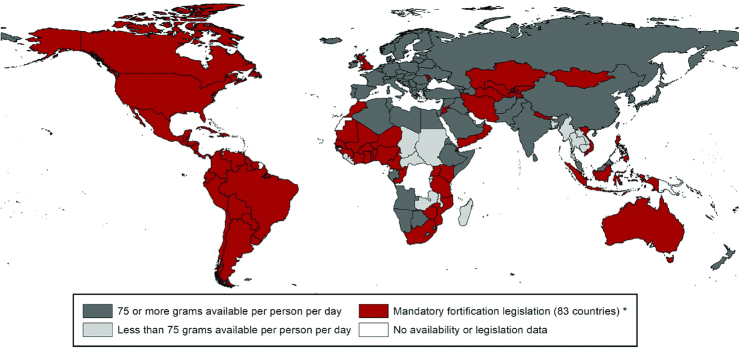
Countries with mandatory wheat flour fortification and those with wheat availability of ≥75 g/d per capita, as of August 2019 [reproduced with permission from Food Fortification Initiative (58)]. *Legislation has the effect of mandating grain fortification with at least iron or folic acid. This does not reflect how much grain is available in that country. Grain availability data from the FAO (2013 data accessed in January 2019). Legislation status from the Food Fortification Initiative (www.FFInetwork.org).

Because the salt was widely accepted by the population, it is unclear why this formulation was discontinued. The most likely explanation is the corrosive nature of sodium acid sulfate and the low pH of the DFS which was reported to decompose the salt distribution bags (46), and would also presumably cause concerns during DFS manufacture. Although FOP, without sodium acid sulfate, could still be considered as a potential iron compound for DFS, FPP would now be considered as preferable to FOP. FPP is widely used in the food industry for color-sensitive infant and dietetic foods, and has higher relative bioavailability than FOP in both rats and humans (59, 60).

## Consumption Patterns and Industrial Landscapes for Different Food Fortification Vehicles

A minimum amount of a staple food or condiment must be consumed daily in order to be effective as an iron fortification vehicle. The estimated daily consumption patterns of cereals, milk, and salt in different world regions used in the current article have been taken from previous reviews. It should be noted, however, that these reviews have used different methodologies to estimate food consumption. Consumption of wheat, maize, and rice (61, 62) are based on food balance data and represent food that is available for consumption, but not necessarily consumed. Milk consumption, on the other hand, was based solely on nationally representative dietary surveys (63), and daily salt intake was estimated based on 24-h sodium excretion and dietary surveys (64).

It is likewise important that the food vehicle selected for fortification is industrially processed and thus potentially fortifiable. The 2 major reasons for poor coverage of current national fortification programs were recently identified as low consumption of the fortification vehicle or that the bulk of the vehicle consumed was not industrially processed and thus not fortifiable (65).

### Wheat and maize

#### Consumption patterns

Wheat and maize are 2 of the most important food crops worldwide. These cereals are grown in Africa, the Americas, Asia, Europe, and Oceania. They are common food staples in many countries from all world regions, with 350 million metric tons (MT) of wheat and 88 million MT of maize available annually for human consumption. Based on food balance data, some countries have >400 g wheat/d per capita available for consumption. These include Azerbaijan, Turkey, Iran, Georgia, Afghanistan, and some North African countries, but on average globally 194 g/d of wheat and wheat products are available per capita for human consumption (61). In contrast, there are only a few countries with a maize availability >200 g/d per capita. These include Malawi, Zambia, Zimbabwe, Kenya, and South Africa in Africa, and Guatemala, Honduras, and Mexico in Central America, but on average globally only 61 g/d per capita of maize and maize products are available for human consumption (61).

**FIGURE 2:**
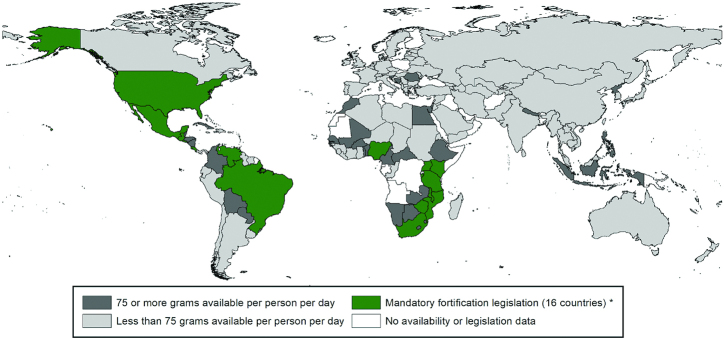
Countries with mandatory maize flour fortification and those with maize availability of ≥75 g/d per capita, as of August 2019 [reproduced with permission from Food Fortification Initiative (67)]. *Legislation has the effect of mandating grain fortification with at least iron or folic acid. This does not reflect how much grain is available in that country. Grain availability data from the FAO (2013 data accessed in January 2019). Legislation status from the Food Fortification Initiative (www.FFInetwork.org).

The WHO (6) has estimated that ≥75 g/d per capita is needed to be able to adequately fortify cereal flour with iron so as to meet the needs of women of child-bearing age. This amount is surpassed in many countries for wheat flour availability (**Figure 1**), but in far fewer for maize flour availability (**Figure 2**), and 161 countries have a combined wheat and maize flour availability that meets this requirement (61).

#### Industrial landscape

The industry landscapes for wheat and maize flour differ in their extent of industrialization. The global wheat flour industry is well established and highly consolidated with ∼80% of wheat flour milled industrially (66). An industrial wheat mill is defined as one with a capacity ≥20 MT/d. A standard industrial mill today, however, would process between 150 and 600 MT wheat/d (68). On the other hand, only 36% of maize is milled industrially with most being milled in small nonindustrial mills (<20 MT/d) (66). Small nonindustrial mills predominate in Africa and Central America, where maize is consumed in greater amounts, although commercial processing of maize in Africa is increasing (61). In 2018, 32% of industrially milled wheat and 54% of industrially milled maize was fortified (66). Mandatory legislation for wheat flour fortification has been introduced in 83 countries (Figure 1) (58) and mandatory legislation for maize flour fortification in 16 countries (Figure 2) (67). Fourteen further countries allow voluntary fortification of wheat flour (69).

The quality of the fortified cereals is carefully monitored using international standards in the large industrial mills (61). These mills can have automated monitoring systems for quality assurance and quality control and can be subject to government inspections and audits. However, small-scale mills often have little process control and, although fortification is possible, the additional cost of premix, quality control, and monitoring decreases its sustainability and the quality of the fortified flour (61).

### Rice

#### Consumption patterns

**FIGURE 3:**
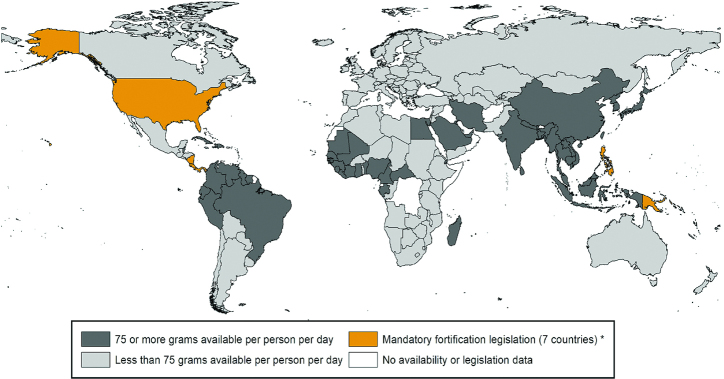
Countries with mandatory rice fortification and those with rice availability of ≥75 g/d per capita, as of January 2019 [reproduced with permission from Food Fortification Initiative (70)]. *Legislation has the effect of mandating grain fortification with at least iron or folic acid. This does not reflect how much grain is available in that country. Grain availability data from the FAO (2013 data accessed in January 2019). Legislation status from the Food Fortification Initiative (www.FFInetwork.org).

Rice is consumed mainly in Asia, although it is becoming an important food staple in both Latin America and Africa. Around 400 million MT rice/y is available for human consumption with China and India accounting for about half the total amount consumed. Almost 50 countries have sufficient rice availability (>75 g/d per capita) for a national iron fortification program (**Figure 3**) (70). These include all countries in Asia, and a large number in Latin America, Africa, and the Middle East. In India, per capita availability is from 100 to 200 g/d per capita compared with 200–300 g/d per capita in China. Rice is available at even higher amounts (>300 g/d per capita) in other Asian countries including Bangladesh, Laos, Cambodia, Vietnam, Myanmar, Thailand, Indonesia, and Philippines. Relatively high per capita availability has also been reported in Latin America and Caribbean countries, with a mean availability of 125 g/d per capita in South America and almost 200 g/d per capita in the Caribbean (62). Urban populations in some West African countries, including Liberia, Mali, Senegal, Gambia, and Côte d'Ivoire, also have rice availability of >200 g/d per capita (62), whereas in much of Europe, North America, and Africa <50 g/d per capita of rice is available. About 90% of the global rice production is in Asian countries.

#### Industrial landscape

The global rice milling industry is relatively well consolidated, with some 56% of rice for human consumption being industrially milled (66). However, because satisfactory rice fortification technologies have been slow to develop, only 1% of industrially milled rice is currently fortified. Seven countries have mandatory rice fortification (Figure 3). Rice fortification is considered financially sustainable in mills where production is >120 MT/d. Although many modern rice mills generally have a much larger capacity, a large proportion of rice is still milled by farmers in thousands of small- and medium-sized mills using old machinery and technology.

Rice is mostly eaten in the country where it is produced and is one of the most protected food commodities. Only 12 countries account for >90% of the global rice trade, and state trading enterprises control import and export of rice in China, India, Indonesia, Japan, South Korea, Vietnam, and Australia. Countries in Latin America, the Caribbean, and Africa are importing increasing quantities of rice (62).

The widespread introduction of fortified rice would need 2 additional steps in the rice supply chain (19). The fortified kernel would need to be manufactured and then blended with the regular rice. These steps could be carried out in the rice mill or at a separate location. Other micronutrients could be added to the fortified kernel if required. Compared with wheat and maize fortification, widespread rice fortification is only beginning to be implemented. Much investment is still required and there is a need to set up legislation and to put into place the rigorous control and monitoring systems used for wheat and maize flours. Muthayya et al. (62) listed several countries which have investigated rice fortification, mostly by extrusion, and supplied fortified rice to public sector programs or retail markets. Whereas industrial mills could more easily cope with the fortification process, the potential to fortify rice in small- and medium-sized village mills would be much lower.

### Milk

#### Consumption patterns

**FIGURE 4:**
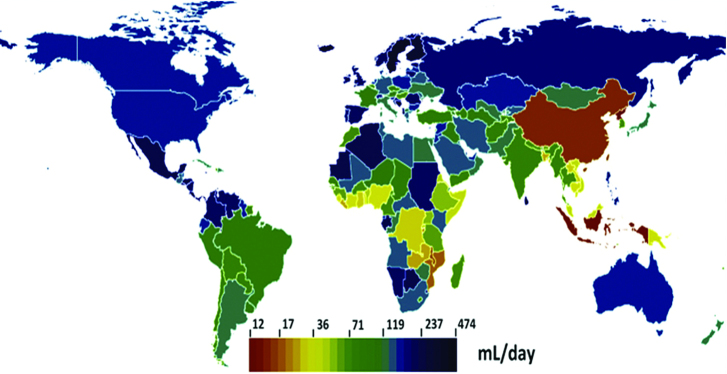
Global milk consumption per country [reproduced with permission from López de Romaña et al. (71)].

Milk is consumed widely around the world, although demand varies considerably from country to country (71). Cow milk is the most prevalent milk but other animal milks (sheep, goat, buffalo, camel) are also popular. A recent review representing 77 countries (63) reported a mean milk intake of 135 mL/d, with the mean intakes being highest in Latin America (250 mL/d), followed by Europe (187 mL/d) and Southern sub-Saharan Africa (177 mL/d). Milk intake was lowest in East Asia (18 mL/d) and Oceania (57 mL/d) (**Figure 4**) (71). Some countries, including Iceland, Sweden, Costa Rica, Finland, and Sri Lanka, have reported remarkably high milk intakes of ∼400 mL/d.

#### Industrial landscape

The milk industry is well established and has a long history of fortification (71). Vitamin A was first added to fluid milk in the United States in the 1940s and to skimmed milk in the 1970s. Several countries have established mandatory fortification of liquid milk with ≥1 micronutrients. Latin America is the region where fortification of milk is most widespread, with whole milk, skimmed milk, and powdered milk often fortified with vitamins A and D. Costa Rica is the only country worldwide, however, that has mandated iron fortification of liquid milk (71). In Costa Rica, both dried and liquid milk are fortified with iron and folic acid. Iron-fortified liquid milk has not been introduced widely elsewhere, presumably owing to the failure to find an alternative iron compound to the very expensive FBG or MDFP (3).

In contrast, powdered milk can be fortified with ferrous sulfate (or ferrous gluconate) and ascorbic acid without unacceptable sensory changes and, although iron-fortified powdered milk has not been used to target ID in the whole population, it is commonly used to target ID in infants and young children (3). In several Latin American countries (Argentina, Chile, Colombia, and Mexico), iron-fortified dried powdered milk is specifically targeted at young children via government-subsidized programs (71). This strategy could also be considered as a means to provide an additional iron supply to adolescent girls and young women planning pregnancy.

### Salt

#### Consumption patterns

The big advantage of salt is its universal consumption at a relatively constant amount in all countries by all population groups independent of socioeconomic status. In most developing countries, where manufactured fortified foods are less widely available, salt is regularly consumed via cooking or with food at meal times, and its intake by adults and children within a specific region or country is very uniform (72). Salt iodization has been implemented in 140 countries, including most countries in Asia, Africa, and Latin America.

Powles et al. (64) reported regional and national salt intakes for adults in 187 countries between 1990 and 2010. Salt intakes were highest (10–13 g/d) in East Asia, Central Asia, Eastern Europe, and South East Asia; followed by 8–10 g/d in North Africa, the Middle East, Central and Western Europe, South Asia, the United States, and Australasia; and by 5–8 g/d in Latin America, Sub-Saharan Africa, the Caribbean, and Oceania. Salt intakes were 10% higher in men than in women and increased only slightly with age. The highest intakes in East and Central Asia and Eastern Europe were thought to be related to salt being more widely used for food preservation.

Although salt intakes were reported to have changed little between 1990 and 2010 (64), there is currently an international effort to decrease salt intakes to <5 g/d as a means to improve cardiovascular health (73). Consumers in China now appear to be following this advice because salt intakes in men and women are reported to have fallen by 4–5 g/d between 1997 and 2011 (74). There is still some way to go for countries to achieve the recommended consumption amounts, however, and 5 g salt/d is still ample for iodine fortification. Iodine has a relatively low EAR of ∼100 μg/d in adults and children (75) and causes no sensory problems. This compares with an EAR of ∼14 mg Fe/d for adolescent boys, adolescent girls, and women of child-bearing age consuming a 10% iron bioavailability diet (3). A reduction in salt consumption would create a need for a higher iron fortification amount in DFS. This could make adverse color formation and iodine losses more likely in DFS, and make iron fortification of salt more difficult.

#### Industrial landscape

Some countries extract their salt from mining of underground rock salt deposits, whereas others extract salt from sea water, saline lakes, or underground brines by solar drying (72). In salt-producing countries, large industrial salt producers are reported to account for nearly 75% of all edible salt consumption (72). However, there still exists a sizable number of small salt producers, especially in coastal areas and along lake shores, who produce lower-quality salt. For example, in Senegal there are >10,000 small salt producers and in the Indian state of Rajasthan small salt producers account for 88% of the total production of salt for human consumption (72). Although quality assurance methods for iodization have been established at the factory and the household level to cover iodine concentrations, packaging quality, and stability of iodine during storage, iodization in small-/medium-sized establishments still poses significant challenges because manufacturing techniques and salt quality vary considerably.

Moorthy and Rowe (76) describe experiences so far with large-scale DFS programs. Only 2 countries, Argentina and India, are producing DFS for the commercial market. In Argentina, DFS Type 5 is produced commercially and is reported to have a yellow coloration; in India, 1 voluntary DFS producer uses DFS Type 3. EFF was approved in India in 2014 and is being manufactured as DFS Type 1c for distribution to government-subsidized social safety net programs.

#### Salt in bouillon cubes, sauces, and processed foods

In some regions of the world, salt may not be directly consumed as salt but be added to, and consumed within, other condiments such as bouillon cubes, fish sauce, and soy sauce, or added to commercially processed foods. Iodized salt may be added to such condiments and processed foods and, should DFS be added in its place, the influence of the different DFS formulations on the sensory properties of bouillon cubes, sauces, and processed foods would need investigation.

Bouillon cubes are a major source of salt for cooking in West Africa, where 1–4 g bouillon · person^−1^ · d^−1^ is consumed on a regular basis (77). The cubes (4 g) contain 40–70% salt together with hardened vegetable fat, hydrolyzed vegetable protein, starch, herbs, spices, and flavorings. Several cubes are usually added daily to the family meal which is cooked in a large pot. The cube is often fortified with iodized salt and iron as FPP or MGFP. Whereas the iodized salt in the cube may make a useful impact on iodine intake (78), the contribution of iron from bouillon (0.6–2.5 mg/d) at present makes only a minor addition to iron intake (77). DFS added to the cubes may thus provide a more useful increment in iron intake.

Soy sauce is the leading condiment in Asian countries with China consuming close to 6 million MT, Japan 900,000 MT, and Indonesia 400,000 MT (79). In Vietnam and Thailand, *c*.300,000 MT of fish sauce is consumed annually. These condiments are affordable, regularly consumed, and widely used as a source of salt. They can be useful sources of iodine (79) if manufactured with iodized salt. Because of their dark color, they are not sensitive to color changes with the addition of iron, and fish sauce and soy sauce fortified with more bioavailable iron compounds, such as NaFeEDTA and ferrous sulfate, have shown good efficacy (79). In some Asian countries, it might be more appropriate to double-fortify fish or soy sauce with iodine and iron as NaFeEDTA rather than to manufacture DFS, although the influence of iron on iodine stability in the sauces would need to be carefully investigated.

## Potential of DFS as Compared with Alternative Iron Fortification Vehicles to Improve Population Iron Status

This section first evaluates the evidence that salt, wheat flour, maize flour, rice, and milk can be effective food vehicles for micronutrient fortification in public health interventions and then compares the potential of each vehicle to improve iron status specifically.

For the comparison, emphasis has been put on the demonstrated efficacy of the respective iron fortification technologies, the consumption patterns of the chosen vehicle, and whether the food industry at the country level is able to rigorously manufacture and control the iron-fortified food. The risk that the current fortification technologies cause unacceptable adverse changes in color or flavor during storage of the fortified food, or during meal preparation, is also considered because this could decrease consumer acceptance and potentially decrease intake and thus impact. Another consideration, specifically for the DFS technology, is whether the addition of iron is likely to increase iodine losses and perhaps jeopardize iodization programs.

### Potential of different fortified food vehicles to decrease micronutrient deficiencies

Salt, wheat flour, maize flour, and liquid milk fortified with specific micronutrients all have a proven record as food vehicles to supply additional micronutrients lacking in the national diet and to decrease or eliminate widespread micronutrient deficiency diseases. In the first half of the 20th century, iodine added to salt, niacin added to cereal flours, and vitamin D added to milk helped to largely eliminate goiter and cretinism, pellagra, and rickets, respectively, in the countries in which the fortification programs were introduced (80). More recently, folic acid added to cereal flours in national programs has greatly decreased the prevalence of neural tube defects (81) and universal salt iodization has decreased the proportion of countries suffering from mild to severe iodine deficiency from 54% in 2003 to 32% in 2011 (82), and to only 17 countries in 2017 (83).

There is good evidence therefore that salt, wheat flour, maize flour, and milk could be effective vehicles for iron fortification. Rice would also be expected to be an effective vehicle for iron fortification; however, because an acceptable technology for micronutrient fortification of rice grains has only recently been introduced, there is as yet no evidence for this from national programs.

Although national iron fortification programs with wheat flour, and to a lesser extent maize flour, have been in place for a considerable time, good evidence of impact on ID at the population level has been difficult to confirm. There are 2 specific reasons for the poor performance of these programs (in addition to the more general reasons for the poor performance of all fortification programs) (65). Firstly, the impact of iron fortification programs has been largely measured based on anemia prevalence which is not a useful iron status biomarker because it is influenced by many other physiological conditions, including infections, inflammation, and hemoglobinopathies common in many LMICs. As a result, iron fortification programs in such countries are reported to result in small, unimpressive decreases in anemia prevalence (84). The second reason is that many earlier national programs fortified wheat flour with nonrecommended elemental iron powders which, based on current knowledge, would not be expected to improve iron status (3).

### Factors influencing the potential of different food fortification vehicles to improve iron status

#### Efficacy

Currently used iron fortification technologies for wheat flour, maize flour, rice, milk, and DFS have all demonstrated a good potential to improve iron status in women and children. However, the fortification technologies for rice and DFS are still under development and further refinements can be expected.

In relation to the currently used DFS formulations, efficacy has only been confirmed with DFS Type 5, and not with DFS Type 2 or DFS Type 1c containing the newly developed EFF. The efficacy of DFS Type 2 and DFS Type 1c thus needs confirmation. A stable isotope iron absorption study is also needed with DFS Type 1c to evaluate the influence of its sophisticated capsule on iron absorption from ferrous fumarate so as to better define the iron fortification amount. Further developments with DFS Type 5 are also possible because it would appear that regular FPP could be used instead of MGFP without decreasing efficacy, and iron absorption enhancers such as trisodium citrate and citric acid could be evaluated.

#### Consumption patterns

Salt is the only vehicle which is universally consumed in relatively constant amounts in almost all countries and by all population groups independent of socioeconomic status. Mean daily salt consumption worldwide is reported to vary from 5 to 13 g and this amount of DFS could theoretically be used to supply iron, as well as iodine, in most countries worldwide. Where soy sauce, fish sauce, and bouillon cubes are consumed as sources of salt, they could potentially be double fortified and used as an alternative to DFS.

Nevertheless, most countries worldwide will have a choice of the iron fortification vehicle. The WHO considers a cereal consumption of >75 g/d per capita to be sufficient for iron fortification (6). Well over 100 countries worldwide have sufficient available wheat flour and >150 countries worldwide have a combined daily availability of wheat and maize flour that is considered adequate, compared with ∼50 countries with sufficient rice availability. Rice consumption is high and widespread in Asia and, with the recent development of the extruded grain premix technology, iron fortification of rice can now be considered for this region, as well as in specific countries in Latin America and Africa where rice intake is also high. Milk is also widely consumed in sufficient quantities in many countries globally, with the exception of Asia and Oceania, and could be a vehicle for iron fortification if an acceptable technology can be developed for liquid milk. Iron-fortified dried milk continues to be a popular vehicle to provide additional iron to young children, especially in public health interventions.

#### Industrial landscape

Iron fortification technologies are relatively simple and iron fortification can be carried out with relative ease with all fortification vehicles, provided fortification is made in large industrial mills or large-scale manufacturing plants. In such establishments, quality control and monitoring of fortification can be carried out according to international standards, with many having automated control systems and regular government audits. Under such conditions, iron fortification is highly likely to improve the iron status of the population. When fortification is carried out in nonindustrial, local mills, or small-scale manufacturing plants, there is usually little process control and less chance of having a fortified food which will improve iron status. The cost of the nutrient premix and quality control in the small-scale mills and manufacturing plants also makes fortification less sustainable.

Approximately 80% of wheat flour is milled in large industrial mills, and large-scale industrial salt producers account for almost 75% of edible salt. This compares to ∼56% of rice, and only 36% of maize, being milled in large industrial mills. The industrial landscape of milk in this respect is not known. Globally ∼20% wheat flour, 25% salt, 45% rice, and 70% maize flour is produced in small-scale, nonindustrial manufacturing plants where there would be much more concern over the quality of iron fortification. Unfortunately, the small-scale local manufacturing plants or mills are usually situated in the populations with the highest risk of iron deficiency. The 70% of maize mills that are small-scale are in the regions of Latin America and Africa with the highest consumption of maize. With respect to DFS, the 25% of producers that are small-scale would also be more likely to produce low-quality DFS which is more susceptible to color changes and iodine losses.

#### Fortification technology

The fortification technologies developed for the different food vehicles are described in more detail by Hurrell (3). They have been developed mainly with the aim of obtaining an adequate iron absorption without causing any sensory changes. The iron fortification technologies developed for wheat flour, maize flour, rice, and milk have all largely eliminated concerns over the formation of adverse color and flavor changes during storage of the iron-fortified food vehicle, or during subsequent processing and meal preparation. However, the optimal extrusion conditions for premix rice have still to be decided, and DFS fortification technology needs further evaluation so as to avoid adverse color development, iron-catalyzed iodine losses, and concerns over consumer safety.

##### Color changes with DFS

In India, where DFS is being manufactured and distributed, salt quality and moisture content vary considerably, as do environmental temperature, humidity, and packaging during DFS storage. It is essential therefore to have a good understanding of how these parameters influence color development in DFS. Although sensory studies have been reported for DFS Types 1b, 2, and 5 in tropical Africa (85), there are no systematic comparisons of currently used DFS formulations made with different-quality salts and stored under different conditions. The black specks observed with DFS Type 1b during storage are thought to be due to salt impurities reacting with ferrous fumarate that has been exposed by abrasion of the capsule during mixing. The newly developed capsule for DFS Type 1c hopefully will prevent this but, as yet, this has not been confirmed. With the further development of DFS technology, further refinements of DFS Type 5 are also possible and the yellow color developed in salt fortified with MGFP can perhaps be prevented by using the larger-particle-size regular FPP, by encapsulating MGFP with a simple hydrogenated fat coating, or by adding SHMP as an iron complexing agent.

There are 2 additional issues related to the development of adverse color formation with DFS. Firstly, if the international recommendations to decrease salt intake are followed, mean salt intakes worldwide could decrease from ∼10 g/d to <5 g/d, creating the need to increase the iron concentration in DFS and thus increasing the potential for adverse color formation. Secondly, because salt is widely added to processed foods such as breads, cheeses, hams, soups, and canned vegetables, and to meals prepared at home, using DFS would carry the risk of causing unacceptable color and flavor changes.

##### Iodine losses with DFS

Although low iodine losses have been reported during the storage of DFS Type 1b and DFS Type 5 made with high-quality dry salt, extensive iodine losses are possible with DFS prepared with low-quality or humid salt, even in the absence of iron. Iodine losses in humid salt are also greater with DFS Type 5. Improved drying and better packaging have markedly decreased iodine losses in iodized salt in recent years, and care should be taken that iodine losses are not again increased by the addition of iron. There is still a need therefore to compare iodine losses with DFS Type 1c, DFS Type 2, and DFS Type 5, made with both MGFP and regular nonmicronized FPP, in different-quality salts stored under different conditions of temperature, humidity, and packaging.

##### Safety of DFS

This issue relates only to DFS Types 1b and 1c where titanium dioxide is used in the capsule to mask the red color of ferrous fumarate. Although this pigment is widely used to enhance the white color and opacity of foods, there has been some concern over the safety of titanium oxide dust inhaled by workers during the manufacturing process. In this respect, the International Agency for Cancer Research concluded that whereas there was inadequate evidence from epidemiological studies to assess whether titanium dioxide dust causes cancer in humans, there was sufficient evidence to conclude that prolonged inhalation in rats causes respiratory tumors (86). More recently, however, concern has been raised over the possible negative health effects of titanium dioxide consumed in food on the gut mucosa and on its associated lymphoid system (87).

## Conclusion

At the present time, in populations where there is adequate consumption of industrially milled wheat flour, wheat flour is technologically preferable for iron fortification. The wheat milling industry is highly industrialized, with a long experience in food fortification. With industrial processing, automatic monitoring of fortification quality to meet international guidelines and government audits are possible. In addition, the fortification technology includes efficacious options for different-quality flours and different environmental conditions. When wheat flour is fortified with NaFeEDTA, it has the additional advantage that it can increase iron absorption from both fortification and native food iron present in the high-phytate diets common in many LMICs. Clearly however, there are countries, or regions of large countries, where wheat flour consumption is not adequate or where wheat is not industrially milled. In these contexts, other food staples or condiments that are sufficiently well consumed should be considered as vehicles for iron fortification.

In many countries or regions with adequate rice or maize consumption, the milling industry is not yet sufficiently industrialized to allow for sustainable fortification. Fifty percent or more of maize and rice grains are milled in small-scale local mills, which do not have the finance or infrastructure to install and control sustainable fortification procedures, and thus will not always be able to ensure an adequately fortified product. In addition, rice fortification technology is still being refined and implementation is at an early stage. Nevertheless, where iron-fortified rice can be produced in large industrial mills, there is ample evidence to conclude that it has a great potential to decrease ID in Asian, African, and Latin American countries with high rice consumption. Liquid milk probably has the least global potential, especially because of its shorter shelf life during storage in the hot and humid climates of some LMICs, but also because a satisfactory, affordable iron fortification compound has not yet been found. However, iron-fortified powdered milk remains an ideal vehicle to improve the iron status of young children.

Several DFS formulations have equally as good iron efficacy as wheat flour, maize flour, or rice, and salt has the advantage of being universally consumed in adequate quantities, with more predictable consumption patterns in all countries worldwide. A disadvantage, relative to the cereals, is the much lower quantity of salt consumed daily, because this necessitates a much higher concentration of iron in DFS than in fortified cereals. Color changes are therefore more likely, especially with the more soluble iron compounds, and the incremental cost to the consumer is higher.

Because almost 75% of salt is manufactured in large industrial mills, iron fortification of high-quality dry salt is possible; however, in many countries there are still many smaller production units manufacturing lower-quality humid salts. However, the DFS technology is not yet fully ready for widespread implementation. Adverse color development is reported during DFS storage, and concerns have been raised over the extent of additional iron-catalyzed iodine losses, and the safety of the titanium dioxide used in the EFF capsules. There is, in addition, no consensus concerning the best iron compound for DFS.

Further developments are therefore needed to be able to manufacture DFS with salts of all qualities, under all storage conditions, without color changes and iodine losses, and at iron concentrations that allow for a salt consumption of 5 g/d. An affordable DFS technology which causes little or no color change, and which results in similar iodine losses to iodized salt (without added iron), can then be considered for national iron fortification programs together with wheat flour, maize flour, and rice. The final choice of food vehicle for a national iron fortification program will then depend on consumption patterns, the industrial landscape, and relative costs.

## Notes

Supported by the Iodine Global Network.

Author disclosures: The author reports no conflicts of interest.

Published in a supplement to *The Journal of Nutrition*. The Supplement Coordinator for the supplement publication was Becky Tsang, Iodine Global Network, Seattle, WA, USA. The Guest Editor for this supplement was Becky Tsang and she has no conflicts of interest. Publication costs for this supplement were defrayed in part by the payment of page charges by the Iodine Global Network. The opinions expressed in this publication are those of the authors and are not attributable to the sponsors or the publisher, Editor, or Editorial Board of *The Journal of Nutrition*.

Abbreviations used: BIS, body iron stores; DFS, double-fortified salt; EAR, Estimated Average Requirement; EFF, encapsulated ferrous fumarate; FBG, ferrous bisglycinate; FOP, ferric orthophosphate; FPP, ferric pyrophosphate; Hb, hemoglobin; ID, iron deficiency; IDA, iron deficiency anemia; LMIC, low- and middle-income country; MDFP, micronized dispersible ferric pyrophosphate; MGFP, micronized ground ferric pyrophosphate; MT, metric tons; NaFeEDTA, sodium iron ethylenediaminetetraacetic acid; PF, plasma ferritin; SF, serum ferritin; SHMP, sodium hexametaphosphate; TfR, transferrin receptor.
